# Podocalyxin is a marker of poor prognosis in colorectal cancer

**DOI:** 10.1186/1471-2407-14-493

**Published:** 2014-07-08

**Authors:** Tuomas Kaprio, Christian Fermér, Jaana Hagström, Harri Mustonen, Camilla Böckelman, Olle Nilsson, Caj Haglund

**Affiliations:** 1 Department of Surgery, Helsinki University Central Hospital, PO Box 440, 00029 Helsinki HUS, Finland; 2 Research Programs Unit, Translational Cancer Biology, University of Helsinki, Helsinki, Finland; 3 Fujirebio Diagnostics AB, Elof Lindälvs gata 13, SE-414 58 Göteborg, Sweden; 4 Onson Consulting, Södra vägen 2, SE-412 54 Göteborg, Sweden; 5 Department of Pathology, Haartman Institute, University of Helsinki and HUSLAB, Helsinki FIN-00014 HY, Finland

**Keywords:** Colorectal cancer, Prognosis, Podocalyxin, Immunohistochemistry

## Abstract

**Background:**

Over two decades ago, a proposal was that two different colorectal cancer (CRC) entities existed, based on tumour location either proximal (right) or distal (left) of the splenic flexure. Proximal and distal tumours exhibit different clinical, epidemiological, and biological characteristics. Improvement of the prognostic evaluation of CRC requires new molecular markers. Podocalyxin-like 1 (PODXL), an anti-adhesive transmembrane sialomucin, is associated with an aggressive tumour phenotype and poor prognosis. For colorectal cancer, it has been suggested to be a marker of poor prognosis. The aim of this study was to investigate the role of PODXL in CRC by use of a novel monoclonal antibody.

**Methods:**

In 1983–2001, 840 consecutive colorectal cancer patients were treated at Helsinki University Central Hospital, of whom 767 were successfully scored for PODXL immunohistochemical expression from tumour tissue microarrays by use of a novel monoclonal in-house antibody. Associations of PODXL expression and tumour location with other clinicopathological variables were explored by Fisher’s exact-test, linear-by- linear association test, and binary logistic regression. Survival analyses were done by the Kaplan-Meier method and Cox proportional hazards model.

**Results:**

PODXL protein expression was high in 44 (5.7%) specimens. High expression associated strongly with poor differentiation (p < 0.0001), advanced stage (p = 0.011), and location of the tumour in the right hemicolon (RHC) (p < 0.001). Tumours of the RHC were more poorly differentiated (p < 0.0001) and showed higher PODXL expression (p < 0.001).

High PODXL expression associated significantly with higher risk for disease-specific death from CRC (hazard ratio (HR) = 2.00; 95% confidence interval (CI) 1.31–3.06, p = 0.001) and also in the subgroups of left hemicolon (LHC) cancers (HR = 2.60; 95% CI 1.45–4.66, p = 0.001) and rectal cancers (HR = 3.03; 95% CI 1.54–5.60, p = 0.001). Results remained significant in multivariable analysis (respectively, HR = 1.82; 95% CI 1.15–2.86, p = 0.01; HR = 2.59; 95% CI 1.41–4.88, p = 0.002; and HR = 2.69; 95% CI 1.30–5.54, p = 0.007).

**Conclusion:**

Podocalyxin was an independent factor for poor prognosis in colorectal cancer and in the subgroups of left hemicolon and rectum. This is, to our knowledge, the first evidence of such difference in PODXL expression, its function possibly being dependent upon tumour location.

## Background

Colorectal cancer (CRC) is the world’s third most common cancer with over one million new cases and half a million deaths annually. Early detection, radical surgical and adjuvant chemotherapy are important for clinical outcome. The most crucial factor today for predicting patient outcome is stage of disease at diagnosis; roughly 40% has localised disease and another 40% regional disease [1].

Over two decades ago, Bufill [2] proposed that two different CRC entities exist according to location of the tumour either proximal to the splenic flexure (RHC = right hemicolon; caecum, ascending colon, hepatic flexure, and transverse colon) or distal to it (LHC = left hemicolon; splenic flexure, descending colon, sigmoid colon, and rectum). Cancers of the RHC (RHCC) and LHC (LHCC) exhibit different clinical and biological characteristics [3-5].

Adjuvant therapy is today standard care for stage-III patients, giving an absolute 10% increase in 5-year overall survival, but for stage-II patients, the benefit of adjuvant therapy is still unclear. In stage-II patients, T4-stage, high histological grade, vascular invasion, tumour obstruction, bowel perforation, and inadequate lymph node resection favour the need for adjuvant therapy, even though limited prospective data support this [6].

Podocalyxin-like 1 (PODXL) is an anti-adhesive transmembrane sialomucin expressed by normal vascular endothelia [7], breast epithelial cells [8], haematopoietic progenitors [9], and renal podocytes [10]. It is also a well-known stem cell marker [11], and is closely related to stem cell marker CD34 and to endoglycan. It is thought to regulate cell morphology and adhesion through its connections to intracellular proteins and to extracellular ligands [12-15]. Aberrant expression or allelic variation of PODXL or both occurs in many cancer forms, including renal cell carcinoma, breast, colorectal, testicular, prostate, and pancreatic cancer [8,13,16-20]. In renal cell carcinoma, breast, and colorectal cancer it has also been an independent predictor of poor prognosis. The role of PODXL is not yet fully understood; though it evidence shows it to participate in epithelial-mesenchymal transition [21] and it interacts with different mediators of metastasis [13-15,20,22].

The aim of this study was to validate in a cohort of 840 CRC patients the role of PODXL expression as a marker of poor prognosis and to evaluate its association with clinicopathological variables by use of a novel monoclonal antibody. This new antibody HES9, produced against embryonic stem cells, is demonstrated to recognise PODXL.

## Methods

### Patients

The study population comprised 840 consecutive colorectal cancer patients surgically treated in 1983–2001 at the Department of Surgery, Helsinki University Central Hospital. Their median age was 66. The Finnish Population Register Centre provided the follow-up vital status data needed to compute survival statistics, and Statistics Finland provided cause of death for all those deceased. Median length of follow-up was 5.1 year (range 0–25.8), with a 5-year disease-specific overall survival rate of 58.9% (95% Cl 55.0–62.8%). The Surgical Ethics Committee of Helsinki University Central Hospital (Dnro HUS 226/E6/06, extension TMK02 §66 17.4.2013) and the National Supervisory Authority of Welfare and Health (Valvira Dnro 10041/06.01.03.01/2012) approved the study.

### Preparation of tumour tissue microarrays

Formalin-fixed and paraffin-embedded tumour samples came from the archives of the Department of Pathology, University of Helsinki. An experienced pathologist marked representative areas of tumour samples on haematoxylin- and eosin- stained tumour slides. Three 1.0-mm-diameter punches taken from each sample were mounted on recipient paraffin block with a semiautomatic tissue microarray instrument (Beecher Instruments, Silver Spring, MD, USA) as described [23].

### PODXL monoclonal antibody

For the novel monoclonal antibody (mAb) HES9 used here, immunization of mice was with the undifferentiated human embryonic (hES) stem cell line SA167 (Cellartis, Gothenburg, Sweden, http://www.cellectis-bioresearch.com); and by conventional hybridoma technology [24] we established hybridoma cell lines producing mAbs against hES cells. Mimotope analysis, immunoprecipitation, and mass-spectrometry identified the target antigen as PODXL. The mimotope sequence corresponds to amino acid residues 189 to 192 in the PODXL protein sequence (NCBI Reference Sequence: NP_001018121.1). For a detailed description see Additional file 1.

### Immunohistochemistry

Tumour tissue microarray blocks were freshly cut into 4-μm sections. After deparaffinization in xylene and rehydration through a gradually decreasing concentration of ethanol to distilled water, slides were treated in a PreTreatment module (Lab Vision Corp., Fremont, CA, USA) in Tris–HCl (pH 8.5) buffer for 20 min at 98°C for antigen retrieval. Staining of sections was performed in an Autostainer 480 (Lab Vision) by the Dako REAL EnVision Detection system, Peroxidase/DAB+, Rabbit/Mouse (Dako, Glostrup, Denmark). Tissues were incubated with the mouse mAb HES9, at dilution of 1:500 (=5 μg/ml) for one hour at room temperature. A sample of renal tissue served as a positive control in each staining series.

### Scoring of samples

HES9 expression in tumour cells was mainly cytoplasmic, evenly distributed, and often granular. Membranous positivity was seen only in cells with strong cytoplasmic staining. Positivity in tumour cells was uniform, with no nuclear expression. Cytoplasmic HES9 immunoreactivity was scored independently by T.K. and J.H., who were blinded to clinical data and outcome. Negative cytoplasmic staining was scored as 0, weakly positive as 1, moderately positive as 2, and strongly positive as 3. The highest score of the triplicates of each sample was considered representative for analysis. Differences in scoring were discussed until consensus.

### Statistical analyses

For statistical purposes, categories of PODXL expression were dichotomised into low (0–2) and high (3). Evaluation of the association between PODXL expression and clinicopathological parameters was done with the Fisher exact-test or linear-by-linear association test for ordered parameters. The effect of laterality on PODXL expression was confirmed by binary logistic regression adjusted for differentiation, age, gender, and Dukes classification. Disease-specific overall survival was counted from date of surgery to date of death from colorectal cancer, or until end of follow-up. Survival analysis was done by the Kaplan-Meier method and compared by the log rank test. The Cox regression proportional hazard model served for uni- and multivariable survival analysis, adjusted for sex, age, Dukes classification, and differentiation. Testing of the Cox model assumption of constant hazard ratios over time involved including a time-dependent covariate separately for each testable variable. Hazard ratios of differentiation and Dukes class D were analyzed in two periods (0 to 1.25 and 1.25 to 5 years) in order to meet the assumptions of the Cox model, and the time-dependent COX model was used. Interaction terms were considered, but no significant interactions found. All test were two-sided. A p-value of 0.05 was considered significant. All statistical analyses were done with SPSS version 20.0 (IBM SPSS Statistics, version 20.0 for Mac; SPSS, Inc., Chicago, IL, USA, an IBM Company).

## Results

### Immunohistochemistry

**Figure 1:**
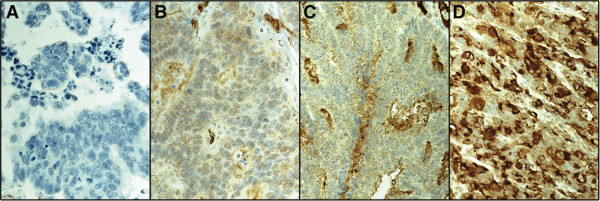
**Immunohistochemical staining patterns of mAb HES9.** Representative images of PODXL expression in colorectal cancer. **(A)** PODXL-negative, **(B)** weakly positive, **(C)** moderately positive, and **(D)** strongly positive immunoreactivity. MAb HES9 recognises PODXL protein. Original magnification was × 40.

Of 840 tumours represented in the TMA, PODXL cytoplasmic staining could be evaluated in 767 (91.3%): 41 (5.3%) negative for PODXL, 430 (56.1%) showing weak staining, 252 (32.9%) moderate staining, and 44 (5.7%) strong staining. Representative immunostainings are in Figure 1A-D. In the vast majority of tumours, PODXL stained evenly throughout the cytoplasm in a granular manner, and positivity was visible in all tumour cells. Neither nuclear nor cell membranous immunopositivity occurred.

### Association of PODXL expression with clinicopathological parameters

Analysis of the association between PODXL and clinicopathological parameters revealed a strong association between high PODXL expression and poor differentiation (p < 0.0001), advanced stage (p = 0.012), and location in the RHC (p < 0.001). PODXL expression did not associate with age, gender, nor tumour location (colon vs. rectum) (Table 1).

**Table 1:** Association between HES9 expression and clinicopathological parameters

**HES9 expression**			
	**Low**	**High**	
**n (%)**	**723 (94.3)**	**44 (5.7)**	**p-value**
**Age, years**			
<65	309 (42.7)	16 (36.4)	0.436
≥65	414 (57.3)	28 (63.6)	
**Gender**			
Male	402 (44.4)	24 (54.5)	1.000
Female	321 (55.6)	20 (45.5)	
**Dukes**			
A	109 (15.1)	2 (4.5)	0.012
B	259 (35.8)	13 (29.5)	
C	196 (27.1)	14 (31.8)	
D	159 (22.0)	15 (34.1)	
**Grade (WHO)**			
1	26 (3.6)	0 (0)	<0.0001
2	514 (71.5)	11 (25.0)	
3	161 (22.4)	25 (56.8)	
4	18 (2.5)	8 (18.2)	
Missing	4		
**Location**			
Colon	372 (51.5)	28 (63.6)	0.123
Rectum	351 (48.5)	16 (36.3)	
**Side**			
Right	189 (26.1)	23 (52.3)	<0.001
Left	534 (73.9)	21 (47.7)	

Fisher exact-test for 2 × 2 tables and linear-by-linear association test for tables with more than two rows. Missing data excluded from analyses. MAb HES9 recognises PODXL protein.

### Association of tumour location with clinicopathological parameters

Analysis of the differences between cancers of the RHC and LHC showed cancers of the RHC to exhibit higher expression of PODXL (p < 0.001) and be more poorly differentiated (p < 0.0001) (Table 2). The effect of laterality on high PODXL expression was confirmed by binary logistic regression adjusted for differentiation, age, gender, and Dukes classification (OR = 2.27; 95% CI 1.17–4.39, p = 0.015). Laterality did not associate with age or gender (Additional file 2).

**Table 2:** Association of tumour side and clinicopathological parameters

**Location of the tumour**			
	**Right hemicolon**	**Left hemicolon**	
**n (%)**	**227 (27.0)**	**613 (73.0)**	**p-value**
**Age, years**			
<65	89 (39.2)	271 (44.2)	0.209
≥65	138 (60.8)	342 (55.8)	
**Gender**			
Male	119 (52.4)	347 (56.6)	0.310
Female	108 (47.6)	266 (43.4)	
**Dukes**			
A	21 (9.3)	104 (17.0)	0.129
B	85 (37.4)	209 (34.1)	
C	70 (30.8)	161 (26.3)	
D	51 (22.5)	139 (22.7)	
**Grade (WHO)**			
1	7 (3.1)	22 (3.6)	<0.0001
2	127 (56.7)	444 (72.7)	
3	74 (33.0)	128 (20.9)	
4	16 (7.1)	17 (2.8)	
Missing	3	2	
**HES9**			
Low	189 (89.2)	534 (96.2)	<0.001
High	23 (10.8)	21 (3.8)	
Missing	15	58	

Fisher exact-test for 2 × 2 tables and linear-by-linear association test for tables with more than two rows. Missing data excluded from analyses. MAb HES9 recognises PODXL protein.

### Survival analysis

**Figure 2:**
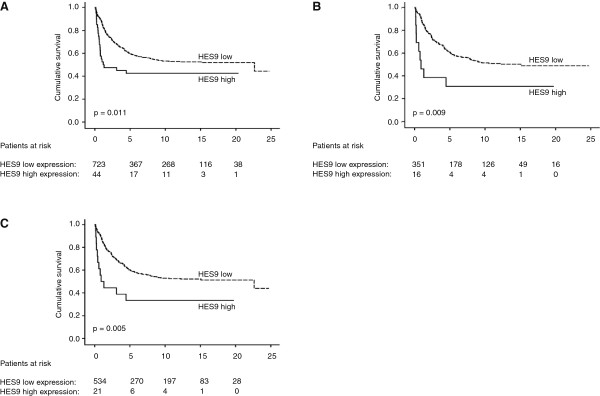
**High expression of PODXL is associated with poor prognosis.** Disease-specific survival analysis according to the Kaplan-Meier method for HES9 expression in **(A)** colorectal cancer, **(B)** rectal cancer, and **(C)** cancer of the left hemicolon. Log-rank test was the test used. MAb HES9 recognises PODXL protein.

Kaplan-Meier analysis showed a significantly poorer disease-specific survival (DSS) for colorectal cancer patients with high PODXL expression (p = 0.011) (Figure 2A). Five-year DSS for patients was 42.5% for high PODXL expression (95% CI 27.2–57.8%) and for low expression 59.8% (95% CI 56.1–63.5%).

Because of the difference in biological and anatomical background of the right and left colorectum, we also stratified our results for colon vs. rectum, and for RHC vs. LHC. For colon and RHC cancer patients we found no evidence of any difference in survival between patients with high compared to low PODXL expression (Additional file 3). Rectal cancer patients with high PODXL expression had significantly poorer DSS than did those with low expression (p = 0.009). Five-year DSS for rectal cancer patients was 30.8% (95% CI 5.7–55.9%) for high and 60.7% (95% CI 55.2–66.2) and for low PODXL tumour expression. A similar effect of high PODXL expression occurred in LHCC (p = 0.005). Five-year DSS for LHCC patients was 33.3% (95% CI 11.5–55.1%) for high vs. 60.2% (95% CI 55.9–64.5%) for low PODXL tumour expression (Figures 2B-C). No significant difference in DSS emerged between tumours having negative, weak or moderate PODXL expression in CRC, or in any of the subgroups (data not shown).

Cox regression univariable analyses confirmed these results. In multivariable survival analyses adjusted for age, gender, Dukes classification, and differentiation grade, high PODXL expression remained significant for the whole CRC material as well as for the subgroups of rectal cancer and cancer of the LHC (Table 3).

**Table 3:** Cox uni-and multivariable analysis of relative risk of death from colorectal cancer, rectal cancer, and left hemicolon cancer within 5 years by HES9 expression

	**Colorectal cancer-specific survival**			**Left hemicolon cancer-specific survival**			**Rectal cancer-specific survival**		
	**HR (95% CI)**	**p-value**	**n (events)**	**HR (95% CI)**	**p-value**	**n (events)**	**HR (95% CI)**	**p-value**	**n (events)**
	Univariable								
HES9 low	1.00		723 (266)	1.00		534 (194)	1.00		351 (125)
High	2.00 (1.31–3.06)	0.001	44 (23)	2.60 (1.45–4.66)	0.001	21 (12)	3.03 (1.54–5.60)	0.001	16 (9)
	Multivariable								
HES9 low	1.00		719 (266)	1.00		532 (194)	1.00		351 (125)
High	1.82 (1.15–2.86)	0.01	44 (23)	2.59 (1.41–4.88)	0.002	21 (12)	2.69 (1.30–5.54)	0.007	16 (9)

*Abbreviations:CI* confidence interval, *HR* Hazard ratio. Multivariable analysis included adjustment for gender, age (>/≤65 years), Dukes class, differentiation grade (G1/2 vs G3/4). MAb HES9 recognises PODXL protein.

## Discussion

Here we used a novel mAb HES9 produced by hybridoma technology against human embryonic stem cells to show that this new mAb is specific for PODXL, known to be a stem cell marker [11]. By immuhistochemical staining, we show that in CRC, PODXL is an independent prognostic factor. CRC has been analyzed as one disease, but here we analyzed separately tumours from the colon and rectum as well as right and left hemicolon. To our knowledge these are the first results to show that PODXL is an independent prognostic factor in subgroups of LHCC and rectal cancers. We also show a difference in PODXL expression depending on tumour location.

We also show that immunostaining of PODXL by our new mAb gives prognostic results similar to those achieved by the commercial polyclonal antibody (HPA 2110, Atlas Antibodies, Stockholm, Sweden) [17,18]. The antigenic determinant of the polyclonal PODXL antibody is amino acid residues 278–415, whereas our antibody reacts with amino acid residues 189–192. One proposal is that distinct membranous PODXL expression, but not cytoplasmic expression, associates with poor prognosis in different cancers [17,25]. Interestingly, staining by our monoclonal antibody was mainly cytoplasmic in cancer cells, with no distinct membranous immunopositivity. The reason for the difference in cancer cells compared to benign cells is not known. The difference in staining pattern may reflect different PODXL function in cancer compared to normal tissue. It is also possible that the HES9 mAb in cancer cells recognices splice variants not expressed in cytoplasm of normal cells. Similar change in expression from membranous to cytoplasmic is also seen for instance in some Toll-like receptor (TLR) stainings [26]. The proportion of tumours with high PODXL expression was relatively small compared to the proportion in previous studies (5.7% vs. 7.9–13.4%) ([17,18], which could be explained by differences in antibody, patient series, staining methods, and staining evaluation/cut- off points. Similar results to ours has been reported in uterine endometroid carcinoma, where the ectopic apical expression of PODXL in benign uterine endometroid tissue was transformed into cytoplasmic expression in carcinoma [27]. Further studies compare the expression pattern of these two antibodies in the same patient series.

High expression of PODXL was an independent marker of poor prognosis in colorectal cancer, but no difference emerged between moderate, low, or negative expression. These results are similar to earlier ones on colorectal [17,18] and breast cancer [8].

Over two decades ago Bufill’s [2] suggestion of subdivision of CRC by tumour location was not based solely on anatomical site. It is based also on developmental differences, because the RHC is derived from midgut and perfused by the superior mesenteric artery with a multilayered capillary network, whereas the LHC is derived from hindgut and perfused by the inferior mesenteric artery with a single layer capillary network.

Here, cancers of the RHC were more poorly differentiated, had higher PODXL expression, and were in older patients, more often female, although differences in age and gender were not statistically significant. Results are in concordance with earlier ones, because cancers of the right hemicolon tend to be less differentiated and more locally advanced, the patients tend to be older, and more often female [4,5,28]. On the other hand, a recent study by Yamauchi [29] suggests no discrete transition point at the splenic flexure, but a gradual change in histological and molecular characteristics from ascending colon to rectum.

The higher expression of PODXL in the RHCC was expected, because these cancers are more poorly differentiated and PODXL expression correlates with differentiation. On the other hand, we show that the difference in PODXL expression between right and left sides is independent of other predictors of survival. Interestingly, only in the LHCC group was high PODXL expression a sign of poor prognosis, not in the RHCC group. This is unlikely due to inadequate statistical power in our study, as there were 237 cases of RHCC, suggesting a different role for PODXL in tumours of the RHC compared to the LHC.

By the TMA technique, only a small proportion of the tumours are evaluated compared to studies of whole tissue sections. Moreover, for technical reasons, up to 9% of the specimens were lost in the TMA-production and -staining process. On the other hand, TMA allows analysis of larger patient cohorts. The strength of this study is a large, well-characterised CRC-patient cohort with long follow-up time that permitted subgroup analyses of patients with various tumour locations.

## Conclusion

These results validate the role of PODXL expression as a prognostic marker in CRC. We also showed the expression and possibly even function not to be universal throughout tumours of the colon and rectum. These findings confirm that CRC should not be considered a uniform disease and indicate the possibilities for more individual diagnosis and treatment. Further studies should aid in understanding the function of PODXL in CRC and the reasons for differences in expression between cancers of the RHC and the LHC.

## Competing interests

No authors have any competing interests.

## Authors’ contributions

TK performed the statistical analyses, participated in data collection, and drafted the manuscript. CF and ON were responsible for production and characterisation of mAb HES9. JH was responsible for scoring of HES9 staining. HM was responsible for statistical analyses. CB participated in data collection and figure design. CH planned the study, was responsible for the immunohistochemical methods, and helped to draft the manuscript. All authors read and approved the final manuscript.

## Pre-publication history

The pre-publication history for this paper can be accessed here:

http://www.biomedcentral.com/1471-2407/14/493/prepub

## Supplementary Material

Additional file 1 PODXL monoclonal antibody. Click here for file

Additional file 2 Effect of clinicopathological parameters on HES9 expression in colorectal cancer. Click here for file

Additional file 3 Cox univariable analysis of risk of death from right hemicolon and colon cancer within 5 years by HES9 expression. Click here for file
