# Supplementary material for: Podocalyxin is a marker of poor prognosis in colorectal cancer
Source: BMC Cancer. 2014 Jul 8;14:493. doi: 10.1186/1471-2407-14-493 (PMC4226963; doi:10.1186/1471-2407-14-493)
Supplement: Additional file 1 — PODXL monoclonal antibody. [file 1471-2407-14-493-S1.doc]

**Additional file 1**

**PODXL monoclonal antibody**

Material and methods

**Production of hybridoma cells**

Conventional hybridoma technology [20] allows establishment of hybridoma cell lines producing monoclonal antibodies (mAbs) against hES cells. Briefly, 8-week-old Balb/c female mice were immunized intraperitoneally with a 100-µl crude whole cell antigen mixture containing approximately 2 × 10^6^ intact undifferentiated hES cells (SA167, Cellartis AB, Gothenburg, Sweden) together with 100 µl of adjuvant Ribi (Corixa Corporation, Seattle, WA, USA). Booster immunizations were performed at regular intervals, ending with a pre-fusion booster three days before fusion. The animals were sacrificed with CO_2_, and the spleens were surgically removed. B-cells from the spleens of immunized mice were fused with the myeloma cell line P3X63Ag8.653 (CRL-1580, purchased from American Type Culture Collection (ATCC), Manassas, VA, USA). To allow efficient screening of a large number of hybridoma supernatants, the primary screening system was designed to contain two different cell types simultaneously, i.e. hES-cells to allow positive screening of potential candidates as well as human foreskin fibroblasts (hFFs) to eliminate candidates with low hES cell specificity. Screening was performed on cells fixed in 4% paraformaldehyde in 96-well plates (Nalge Nunc International; Rochester, NY, USA). The secondary screening was also on hES cells allowed to differentiate spontaneously to select only hybridomas with specificity towards undifferentiated hES cells. All animal experiments in this study were performed in accordance with the Animal Welfare Ordinance and the Animal Welfare Act of Sweden and approved by the animal experiments ethics committee of Gothenburg (approval no. 310-2005).

**Cloning of hybridomas**

The hybridoma cells were cloned by a single-step procedure under visual control. Briefly, the hybridoma cells were diluted in medium to a concentration of 10^4^ cells/ ml. Small drops of the cell suspension (0.4-0.5 μl) were placed on the bottom of 96-well plates and examined microscopically. Hybridoma culture medium was added only to wells confirmed to contain one single cell. This procedure ensures that hybridoma growth is initiated clonally from one cell. Cloning resulted in several subclones; these were screened as described above. The isotypes were determined using HRP-conjugated isotype-specific antibodies (Zymed, South San Francisco, CA, USA) in an ELISA assay.

**Epitope analysis using random peptide libraries displayed on phage**

Two libraries, Ph.D. 7 and Ph.D. 12, display 7 and 12 amino acid randomized peptides, on the surface of phage M13 (New England Biolabs, Hertfordshire, UK). The HES9 mAb was coated on Maxisorp plates and used to pan the Ph. D. 7 or Ph. D. 12 libraries. Four to six rounds of pannings were performed according to manufacturer’s instructions. Individual clones were amplified and sequenced by to standard techniques. Sequence analysis was performed to identify consensus sequences.

Results

**Characterization of mAb HES9 antigen**

MAb HES9 (available from Cellartis AB under the product name ES-Cellect) was selected for high specificity for undifferentiated hES cells and showed loss of reactivity early upon cell differentiation. Mimotope analysis with the 7- and 12-mer libraries identified the mimotope sequence [-PRQP-]. Database searches using the mimotope sequence identified a limited number of proteins with the corresponding epitopes. Taking into account the high expression on vascular endothelium and renal podocytes, the target antigen could be identified as Podocalyxin-like protein 1 (PODXL). The mimotope sequence corresponds to amino acid residues 189 to 192 in the PODXL protein sequence (NCBI Reference Sequence: NP_001018121.1). Immunoprecipitation and subsequent mass-spectrometry analysis could confirm the identity of the target antigen.
