# Supplementary material for: Podocalyxin is a marker of poor prognosis in colorectal cancer
Source: BMC Cancer. 2014 Jul 8;14:493. doi: 10.1186/1471-2407-14-493 (PMC4226963; doi:10.1186/1471-2407-14-493)
Supplement: Additional file 2 — Effect of clinicopathological parameters on HES9 expression in colorectal cancer. [file 1471-2407-14-493-S2.doc]

**Additional file 2**

Effect of clinicopathological parameters on HES9 expression in colorectal cancer

| **Clinicopathological parameters** | **Reference category** | **p-value** | **OR** | **95 % CI for OR** | |
| --- | --- | --- | --- | --- | --- |
|  |  |  |  | **lower** | **upper** |
| Tumour side, right | Left | 0.015 | 2.27 | 1.18 | 4.39 |
| Dukes Class | A | 0.303 | 1.20 | 0.85 | 1.71 |
| Differentiation | High | >0.0001 | 4.40 | 2.71 | 7.12 |
| Gender | Male | 0.73 | 1.12 | 0.58 | 2,17 |
| Age | Under 65 | 0.356 | 1.37 | 0.70 | 2.69 |
|  |  |  |  |  |  |

OR=Odds Ratio. MAb HES9 recognises PODXL protein. Interaction terms were considered, but no significant interactions found.
