# Supplementary material for: Podocalyxin is a marker of poor prognosis in colorectal cancer
Source: BMC Cancer. 2014 Jul 8;14:493. doi: 10.1186/1471-2407-14-493 (PMC4226963; doi:10.1186/1471-2407-14-493)
Supplement: Additional file 3 — Cox univariable analysis of risk of death from right hemicolon and colon cancer within 5 years by HES9 expression. [file 1471-2407-14-493-S3.doc]

**Additional file 3**

**Cox univariable analysis of risk of death from right hemicolon and rectal cancer within 5 years by HES9 expression**

|  | **Right hemicolon cancer-specific survival** | | | **Colon cancer-spesific survival** | | |
| --- | --- | --- | --- | --- | --- | --- |
|  | **HR (95% CI)** | **p-value** | **n (events)** | **HR (95% CI)** | **p-value** | **n (events)** |
|  | Univariable |  |  |  |  |  |
| HES9 low | 1.00 |  | 189 (72) | 1.00 |  | 372 (141) |
| high | 1.51 (0.80-2.84) | 0.204 | 23 (11) | 1.59 (0.91-2.74) | 0.103 | 28 14) |

MAb HES9 recognises PODXL protein.
